# Cognitive functioning in untreated glioma patients: The limited predictive value of clinical variables

**DOI:** 10.1093/neuonc/noad221

**Published:** 2023-12-01

**Authors:** Sander M Boelders, Karin Gehring, Eric O Postma, Geert-Jan M Rutten, Lee-Ling S Ong

**Affiliations:** Department of Neurosurgery, Elisabeth-TweeSteden Hospital, Tilburg, The Netherlands; Department of Cognitive Sciences and AI, Tilburg University, Tilburg, The Netherlands; Department of Neurosurgery, Elisabeth-TweeSteden Hospital, Tilburg, The Netherlands; Department of Cognitive Neuropsychology, Tilburg University, Tilburg, The Netherlands; Department of Cognitive Sciences and AI, Tilburg University, Tilburg, The Netherlands; Department of Neurosurgery, Elisabeth-TweeSteden Hospital, Tilburg, The Netherlands; Department of Cognitive Sciences and AI, Tilburg University, Tilburg, The Netherlands

**Keywords:** cognitive function, glioma, individual predictions, machine learning, precision medicine

## Abstract

**Background:**

Previous research identified many clinical variables that are significantly related to cognitive functioning before surgery. It is not clear whether such variables enable accurate prediction for individual patients’ cognitive functioning because statistical significance does not guarantee predictive value. Previous studies did not test how well cognitive functioning can be predicted for (yet) untested patients. Furthermore, previous research is limited in that only linear or rank-based methods with small numbers of variables were used.

**Methods:**

We used various machine learning models to predict preoperative cognitive functioning for 340 patients with glioma across 18 outcome measures. Predictions were made using a comprehensive set of clinical variables as identified from the literature. Model performances and optimized hyperparameters were interpreted. Moreover, Shapley additive explanations were calculated to determine variable importance and explore interaction effects.

**Results:**

Best-performing models generally demonstrated above-random performance. Performance, however, was unreliable for 14 out of 18 outcome measures with predictions worse than baseline models for a substantial number of train-test splits. Best-performing models were relatively simple and used most variables for prediction while not relying strongly on any variable.

**Conclusions:**

Preoperative cognitive functioning could not be reliably predicted across cognitive tests using the comprehensive set of clinical variables included in the current study. Our results show that a holistic view of an individual patient likely is necessary to explain differences in cognitive functioning. Moreover, they emphasize the need to collect larger cross-center and multimodal data sets.

**Key Points**

- Our comprehensive set of clinical variables fails to reliably predict cognitive functioning.
- A multi-parametric view of individual patients is likely necessary.
- Larger cross-center and multimodal data sets are needed.

**Importance of the Study**

Preoperative cognitive functioning is increasingly taken into account to determine the treatment of choice in view of a personalized onco-functional balance and to better counsel patients. Many clinical variables have been significantly related to cognitive functions before surgery in patients with a glioma. Importantly, the statistical significance of a variable in relation to a cognitive function provides information about the strength of the underlying relationship but does not imply that such variables allow for accurate prediction. Therefore, we may be unable to infer cognitive functioning for (yet) untested patients. The current study shows that the relevance of clinical variables is limited when predicting the cognitive functioning of individual patients. Therefore, clinicians should be cautious to infer cognitive functioning from such variables. Moreover, our results suggest that a holistic view of individual patients may be necessary and stress the need for larger cross-center and multimodal data sets. Last, we hope our study serves as a stepping stone toward predicting cognitive functioning after surgery.

Cognitive impairments are common among patients with glioma before surgery.^1^ It is frequently reported as a great burden by both patients and caregivers,^2^ leading to decreased quality of life,^3^ decreased functional independence,^4^ and impaired medical decision-making capacity.^5^ These cognitive impairments are likely caused by a combination of the tumor’s local and global effects on brain functioning^6^ and are influenced by patient characteristics^7^ and genetic markers.^8^ Unfortunately, the exact causal mechanisms by which primary brain tumors affect different cognitive functions are poorly understood.

Previous work identified many clinical variables to be significantly related to cognitive performance in untreated patients with a glioma. We describe 4 types of clinical variables. First, variables describing the tumor in terms of its size,^9–11^ histology/WHO grade,^8,9,11–13^ and location^9,14,15^ have been related to cognitive functioning. Second, patient characteristics including genetic factors,^8^ age,^7,16^ education,^7^ and to a certain extent sex^16^ have been related to cognitive functioning. Third, the use of medication such as antiepileptic drugs (AEDs) has been related to cognitive functioning in patients^9,17^ and the short-term beneficial effects of corticosteroids are well known in clinical practice.^18^ Last, multiple clinical or patient-reported functional or overall health outcome measures have been related to cognitive function such as the Karnofsky performance scale, American Society of Anesthesiology (ASA) scores,^16^ depression and anxiety questionnaire scores,^19^ and other complaints/symptoms.^20^

Preoperative cognitive functioning is increasingly taken into account to determine the treatment of choice in view of a personalized onco-functional balance^21^ and to better counsel patients (ie determine medical decision-making capacity^5,22^ and help to satisfy patients information needs^23^). Given the large number of predictors described in the literature, one may be inclined to assume that preoperative cognitive functioning can be inferred from clinical variables. Previous research, however, only performed *explanatory modeling* in which one aims to find evidence for a hypothesis regarding a theoretical construct from the observed data.^24^ This often took the form of testing the significance of hypothesized predictors in a uni- or multi-variate linear or rank-based model. Importantly, an explanatory model that accurately describes underlying relationships does not necessarily imply that this model can make accurate predictions for individual patients. Therefore, we may be unable to infer cognitive functioning for (yet) untested patients from clinical variables.

In the current study, the goal is to test if preoperative cognitive functioning can be inferred from clinical predictors. This is tested by performing *empirical prediction*, that is given a set of input variables the prediction of an output value for a new observation.^24^ Much like how good explanatory models do not always allow for good prediction, relationships found by good predictive models are not always significant when tested using explanatory modeling. Explanatory modeling and empirical prediction, however, do complement one another. For a more extensive discussion on the value of predictive modeling and its application in research on stroke, we refer to Bonkhoff.^25^

In addition to not considering empirical prediction, previous studies have 2 other limitations. First, they exclusively used linear regression or rank-based methods. Linear models, however, cannot accurately fit nonlinear relationships when not a priori defined, and both linear models and rank-based approaches are unable to find interactions when not a priori defined. The relationship between predictors and cognitive functioning, however, may be more complex than can be captured by linear or rank-based models. For example, premorbid IQ may protect language function by moderating the effects of lesion volume.^26^ Therefore, using models that can capture nonlinear relationships and interactions may result in more accurate predictions when compared to traditional models.

Second, previous studies only used a small number of variables, even though many variables potentially influence cognitive function. Machine learning models can perform regularization which constrains the complexity of a model, allowing for the use of more variables when compared to traditional models without overfitting.

In this study, we tested how well cognitive functioning can be predicted using a comprehensive set of clinical variables including demographics, tumor characteristics, medicine use, reported symptoms, and functional performance scores. To this end, we trained frequently used machine learning models for 4 different objectives: (1) predicting impairment on at least 1 cognitive test, (2) predicting the number of tests on which a patient is impaired, (3) predicting impairment for each test separately, and (4) predicting cognitive function for each test separately.

## Methods

### Participants

Participants were 340 patients with grade 2, 3, and 4 gliomas who underwent surgery at the Elisabeth-Tweesteden Hospital, Tilburg, The Netherlands, and underwent preoperative cognitive screening as part of clinical care between 2010 and 2019. Patients were not included when their age was under 18, when they had a progressive neurological disease, when they had a psychiatric or acute neurological disorder within the past 2 years, or when they had reduced testability for the neuropsychological assessment. The current patient sample is described (in part) in previous studies.^27–36^ For normative purposes, data from healthy Dutch adults were used.^37,38^ This project was part of a study protocol registered at the Medical Ethics Committee Brabant (file number NW2020-32).

### Material

Patients provided informed consent before a standardized interview was performed to obtain demographic variables such as age, sex, and education (the Dutch Verhage scale). Moreover, measures for anxiety and depression were collected using the Dutch translation of the Hospital Anxiety and Depression Scale (HADS).^39^

Cognitive screening was done using the computerized CNS Vital Signs (CNS VS)^40^ test battery. The psychometric properties of this battery were shown to be similar to the pen-and-paper tests in pediatric patients,^41^ and in patients with various neuropsychiatric disorders and healthy participants.^42^ A well-trained technician (neuropsychologist or neuropsychologist in training) instructed patients before starting each test and reported the test validity within the test battery afterward. Requirements for a test to be valid include the patient understanding the test, showing sufficient effort, having no vision or motor impairments that affect the task, and the absence of any distractions. Invalid tests were excluded from the current study on a test-by-test basis. The CNS VS test battery as administered in our clinical practice included CNS VS its 7 core tests and took approximately 30–40 min to complete.

### Cognitive Test Measures and Standardization

Eight test scores were calculated from the CNS VS results according to the formulas presented in Appendix 1. The resulting scores were converted to sociodemographically adjusted *z*-scores. This was done by correcting for effects of age, sex, and education as found in a sample of normative controls using a multiple regression approach, the same as done in work by Rijnen et al.^37^ on this data set. Test scores were further normalized relative to healthy participants, where scores of healthy participants were set to have zero mean and unit variance. Patients were defined as impaired on a test score when their normalized score was below −1.5 (SD).

### Clinical Characteristics

Variables used for prediction collected from patients’ electronic medical files comprised comorbidities, tumor grade classified according to the WHO guidelines,^43^ histological diagnoses (based on cell origin/molecular markers), IDH mutation status (tested using immunohistochemistry, sequencing, or both), involved hemisphere, use of AEDs, comorbidities, and presenting symptoms. Note that we used the measured values for histological diagnosis, IDH mutation status, and tumor grade in the current study while they can merely be *estimated* preoperatively.^44^

The presenting symptoms were the symptoms recorded during the first consultation with the neurosurgeon and were categorized into 5 broad categories: behavioral/cognitive problems, language problems, epilepsy/loss of consciousness, motor deficits, and headache. For patients aged 55 or older with a WHO grade 4 glioblastoma, the IDH mutation status is not always tested in our clinical practice. As the incidence rate of IDH mutant gliomas in this group is low,^45–47^ missing IDH mutation statuses for this group were assumed to be wild-type. Additional information regarding the imputation of IDH statuses can be found in Appendix 2.

### Tumor Volume and Location

All available anatomical MRI scans (T1, T1 contrast, T2, Flair) were registered to MNI space using affine transformation. Tumor regions were defined as the FLAIR hyperintense region for low-grade gliomas and the T1 contrast hyperintense region for high-grade gliomas and were segmented using a convolutional neural network with a U-Net architecture.^48,49^ All automatic segmentations were manually validated and incorrect segmentations were redone semi-automatically. Voxel-wise tumor volume was calculated from the segmentations. Location was calculated as the percentage of overlap of the segmentations with the 4 lobes using the MNI lobe atlas and was calculated separately for each hemisphere. Details on image registration and segmentation can be found in Appendix 3.

### Machine Learning Models

*Variable Reduction.—*The initial set of variables used for predictive modeling comprised age, sex, education, histological diagnosis, WHO grade, IDH mutation status, tumor lateralization, tumor location, tumor volume, ASA score, presence of comorbidity, corticosteroid use, AED use, anxiety level, depression level, and presenting symptoms. Although machine learning models can deal with many variables, too many variables may negatively affect their ability to make predictions. Therefore, reducing the number of variables is a standard procedure in machine learning.^50^

In what follows, we describe the reduction of variables in 2 steps. First, categories including fewer than 10% of patients were combined given that the resulting category was interpretable and categorical variables were dummy-coded. Second, variables were combined when they showed all of the following: high multicollinearity defined as a variance inflation factor (VIF) > 5, a strong correlation with another variable (*r* > 0.6), and would result in an interpretable combined variable. To identify to-be-combined variables, correlations between each pair of variables were clustered using hierarchical clustering and visualized, and VIFs were evaluated. To prevent certain variables from contributing more to a combined variable, individual variables were normalized with zero mean and unit variance before being combined. Thresholds for both the VIF score and correlations were set based on preliminary experiments and fall within normal ranges of high multicollinearity and moderate correlation.^51,52^

*Model Training and Validation.—*Machine learning models were trained for 4 different objectives.

1. Predicting if the patient is impaired on at least 1 cognitive test (1 model with a dichotomous outcome).
2. Predicting the number of tests on which a patient is impaired (1 model with a continuous outcome).
3. Predicting separately for each cognitive test if a patient is impaired (8 models with dichotomous outcomes).
4. Predicting a patient’s cognitive function separately for each cognitive test (8 models with continuous outcomes).

Objectives 1 and 2 are dependent on the test scores of all 8 cognitive tests. For objectives 3 and 4, models were fit individually for each of the 8 cognitive tests. This results in a total of 18 outcome measures across the 4 objectives.

Thirteen different frequently used machine learning models were evaluated which were selected to span a broad set of characteristics such as their ability to perform regularization, capture nonlinearities, and capture interaction effects. Models were (Logistic) Regression: linear model, ElasticNet: linear model with regularization (L1 and L2), Gaussian Processes: learns a distribution over (complex) functions, Bayesian Ridge: Bayesian linear model with L2 regularization, Bayesian ARD: Bayesian sparse linear model with L1 regularization, K-Nearest Neighbors: predicts based on most similar data points, Decision Tree: simple, interpretable tree-based model, Random Forest: ensemble of decision trees, Support Vector Machines: separates classes or fits data with hyperplanes, can use kernels, XGB Tree: boosted decision trees, builds decision trees sequentially, XGB Linear: similar to XGB Tree but uses linear models, Partial Least Squares: finds new features as linear combinations of original features, Gaussian Mixture Model: classifies based on a mixture of Gaussian distributions.

Models were optimized for, and evaluated according to, the f1 score (harmonic mean between precision and recall) and *R*^2^ score for dichotomous and continuous outcome measures, respectively. Repeated nested cross-validation was used to obtain robust and unbiased performance estimates while also optimizing hyperparameters.^53^ Details on the training process including the repeated nested cross-validation procedure are described in Appendix 4. A list of models used including model characteristics, and hyperparameters to be optimized is provided in Appendix 5. Moreover, more detailed explanations of each model including pros and cons are provided in Appendix 6.

*Interpretation.—*The average performances (f1 or *R*^2^) of the different models resulting from their cross-validation procedures were compared with determine which model performed best for each outcome measure. For models with a dichotomous outcome, performance was compared against a baseline model making uniform random predictions instead of using any of the variables. For models with a continuous outcome, performance was compared against a baseline model making constant predictions which always results in an *R*^2^ of 0. Moreover, resulting hyperparameters as optimized by the training procedure were reported.

In addition to reporting the average performances, the standard deviations in performance among the train-test splits were reported. This was done as in some splits, the performance may be worse than the performance of the baseline model even though on average over all splits the model performs better than the baseline model. To aid interpretation, we adopt the definition that a model results in “*reliable predictions*” when its improvement over the baseline model is larger than the standard deviation of its train-test splits performances. This definition is motivated by the need for a model that consistently performs better than the baseline model regardless of the train-test split. This definition further serves as a threshold for model interpretation as described in the next paragraph, preventing us from interpreting models that may have overfitted on the training data.

Optimized hyperparameters were interpreted for the best-performing models that provide reliable predictions to understand the behavior of the model including the type and amount of regularization. Moreover, Shapley additive explanations (SHAP; Lundberg & Lee, n.d.) were calculated and interpreted for the same models to find the importance of individual variables when making predictions and to explore interaction effects as captured by the models. All code for the analyses made in this study is available as Supplementary Material.

## Results

### Descriptive Statistics

Models were evaluated for 340 patients with a glioma of whom 63.2% had a glioblastoma, 13.2% had an oligodendroglioma, and 23.5% had an astrocytoma. Patients were on average 53 years old and 65.8% of the patients were male. The average test score on measures of cognitive function ranged between −1.62 and −0.35 and impairment ranged between 18.7% and 31.9% depending on the cognitive test.

Missing variables comprised 27.6% of the IDH mutation statuses, 5.6% of the HADS questionnaire scores, and 2.1% of the tumor segmentations and thus tumor sizes and locations. Tumor segmentations were missing for 5 patients with a low-grade glioma as the FLAIR scan necessary for segmentation was unavailable and for 2 patients with a high-grade glioma for which the T1c scan was unavailable. Automatic segmentations were corrected for 19 out of 333 (5.71%) patients. Invalid CNS VS test scores ranged between 0 and 7.4%. These test scores generally were missing when participants did not understand or could not execute the instruction of a (more complex/difficult) test. Moreover, at least 1 CNS VS test score was missing for 21.2% of patients, reducing the number of patients to 268 for objectives 1 and 2. Sample characteristics are provided in Table 1.

**Table 1.** Sample Characteristics

Variable name	Count	Mean/%	Std	Min	25%	50%	75%	Max	Missing (%)
Age	340	53.21	14.34	18	45	55	64	81	0.00
Education	340	5.05	1.14	1	4	5	6	7	0.00
Sex (men)	340	65.88%							0.00
Astrocytoma	340	23.53%							0.00
Glioblastoma	340	63.24%							0.00
Oligodendroglioma	340	13.24%							0.00
WHO grade 2	340	27.65%							0.00
WHO grade 3	340	9.41%							0.00
WHO grade 4	340	62.94%							0.00
IDH1 mutation status (mutant)	246	43.50%							27.65
Lateralization left	340	41.76%							0.00
Lateralization right	340	59.71%							0.00
Frontal lobe left (mm^3^)	333	8 531.99	20 832.66	0	0	0	5 936	164 182	2.06
Occipital lobe left (mm^3^)	333	791.27	3 861.01	0	0	0	0	33 466	2.06
Parietal lobe left (mm^3^)	333	1 860.60	6 135.62	0	0	0	11	42 487	2.06
Temporal lobe left (mm^3^)	333	4 353.59	12 228.73	0	0	0	0	74 049	2.06
Frontal lobe right (mm^3^)	333	9 243.12	18 310.81	0	0	166	9 564	99 580	2.06
Occipital lobe right (mm^3^)	333	987.62	4 653.98	0	0	0	0	43 885	2.06
Parietal lobe right (mm^3^)	333	5 139.08	13 397.25	0	0	0	1 081	77 898	2.06
Temporal lobe right (mm^3^)	333	7 534.59	16 717.71	0	0	0	2 731	93 323	2.06
Tumor size (mm^3^)	333	52 553.37	43 485.95	305	22 153	42 176	71 417	264 510	2.06
ASA I	338	44.97%							0.59
ASA II	338	49.70%							0.59
ASA III	338	5.33%							0.59
Comorbidity	340	47.65%							0.00
Corticosteroid use	340	59.41%							0.00
Antiepileptic drug use	340	48.82%							0.00
HADS anxiety	321	6.58	4.59	0	3	6	10	19	5.59
HADS depression	321	4.66	3.72	0	2	4	7	17	5.59
Presents with attention, executive function, memory, and/or behavioral problems	340	22.35%							0.00
Presents with language problems	340	15.29%							0.00
Presents with loss of consciousness	340	42.94%							0.00
Presents with motor deficits	340	23.24%							0.00
Presents with headache	340	23.82%							0.00
Cognitive test scores									
Verbal memory recognition	323	19.50%	1.22	−3.24	−1.31	−0.35	0.42	2.04	5.00
Visual memory recognition	340	24.71%	1.58	−3.94	−1.46	−0.32	0.77	2.36	0.00
Finger tapping test	315	29.52%	1.37	−4.28	−1.73	−0.74	0.10	2.68	7.35
Symbol digit coding	340	31.47%	1.43	−3.82	−1.79	−0.75	0.20	2.36	0.00
Simple reaction time	334	31.94%	2.21	−7.68	−2.07	−0.51	0.30	1.47	1.76
Stroop interference	315	18.73%	1.40	−3.64	−1.08	−0.26	0.66	2.79	7.35
Shifting attention task	315	23.49%	1.09	−2.35	−1.45	−0.71	0.17	2.96	7.35
Continuous performance test	340	27.65%	1.47	−4.25	−1.61	−0.61	0.32	2.15	0.00
Verbal memory recognition (impaired)	323	2.89							5.00
Visual memory recognition (impaired)	340	76.87%							0.00
Finger tapping test (impaired)	315	19.50%							7.35
Symbol digit coding (impaired)	340	24.71%							0.00
Simple reaction time (impaired)	335	29.52%							1.47
Stroop interference (impaired)	315	31.47%							7.35
Shifting attention task (impaired)	315	31.94%							7.35
Continuous performance test (impaired)	340	18.73%							0.00
Number of impaired scores	268	23.49%	2.78	0	1	2	5	10	21.18
Any score is impaired	268	27.65%							21.18

*Note*: Patient characteristics including cognitive test scores. Cognitive test scores are represented as sociodemographically adjusted *z*-scores and are scaled relative to healthy participants. Patients were defined as impaired on a test score when their normalized score was below −1.5 (SD).

Two patients over age 55 with a grade 4 glioblastoma who were tested for IDH mutation status had an IDH mutant tumor. The other 73 of these patients had an IDH wild-type tumor. For 71 patients over age 55 with a grade 4 glioblastoma, the IDH mutation status was not tested. These were set to wild-type as described in the “Methods” section. After this, only 23 (6.8%) of the IDH statuses remained missing.

### Variable Reduction: Categories

Tumors were generally located in only the right (*n* = 198) or left (*n* = 137) hemisphere. Only 5 patients had a bilateral tumor and were excluded from further analysis.

Only 32 patients had a grade 3 tumor. Tumor grade, therefore, was combined into grade 2 (*n* = 91) and grade 3 + 4 (*n* = 244).

ASA scores were generally I (*n* = 149) or II (*n* = 166). Few patients had an ASA score of III (*n* = 18) and no patients had an ASA score of IV or V. For this reason, ASA was combined into ASA I (*n* = 149) and ASA II + ASA III (*n* = 315).

### Variable Reduction: Collinearity

**Figure 1.**
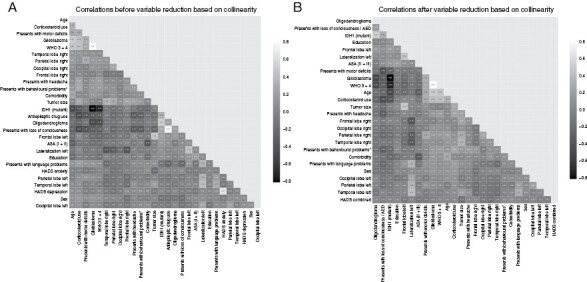
Correlation between variables before (**A**) and after (**B**) reducing the number of variables based on correlations and VIF scores. Variables are clustered according to their correlations.

Correlations between variables before combining variables based on collinearity and correlations are shown in Figure 1A, and VIF scores are displayed in Appendix 7.

Use of AEDs and presenting with loss of consciousness correlated with 0.78 and had high VIF scores of 5.57 and 5.54, respectively. As loss of consciousness is often the result of an epileptic insult, these variables were combined into a variable describing either AED use and/or having presented with loss of consciousness.

Anxiety and depression scores resulting from the HADS had VIF scores of 6.17 and 5.42, respectively, and a correlation of 0.67. For this reason, they were normalized and combined into 1 score.

Glioblastoma and having a high WHO Grade (3 or 4) had a high VIF score of 12.82 and 11.71, respectively, and a correlation of 0.81. Given that the distinction between grade 2 or 3 tumors is informative for both astrocytomas and oligodendrogliomas, we did not combine glioblastoma and high WHO grade.

All other variables with a high VIF did not have a high correlation with any other variable. The final set of variables used for prediction consisted of age, sex, education, tumor size, tumor location (tumor overlap with the 4 lobes separately for each side), lateralization (left vs right), tumor grade (low vs high), histopathological diagnosis (oligodendroglioma, astrocytoma, or glioblastoma), IDH mutation status, presenting symptoms (behavioral/cognitive problems, language problems, motor deficits, and headache), corticosteroid use, use of an AED or loss of consciousness, presence of comorbidity, ASA score (ASA I vs ASA II + ASA III), and the combined anxiety and depression score. Statistics of the final set of variables including VIF scores are presented in Table 2. Correlations between the variables are shown in Figure 1B.

**Table 2.** Descriptive Statistics and VIF Scores for the Variables As Used for Prediction

Variable name	Count	Mean/%	Std	Min	25%	50%	75%	Max	VIF	Missing (%)
Age	335	53.47	14.21	18	45.5	56	64	81	19.54	0.00
Education	335	5.04	1.15	1	4	5	6	7	14.60	0.00
Sex (men)	335	65.97%							3.08	0.00
Glioblastoma	335	63.58%							12.85	0.00
Oligodendroglioma	335	13.43%							1.83	0.00
WHO 3 + 4	335	72.84%							11.46	0.00
IDH1 mutation status (mutant)	312	32.37%							4.03	6.87
Lateralization left	335	40.90%							5.79	0.00
Frontal lobe left (mm^3^)	328	8 110	20 263	0	0	0	5 088	164 182	9.05	2.09
Occipital lobe left (mm^3^)	328	803	3 889	0	0	0	0	33 466	1.50	2.09
Parietal lobe left (mm^3^)	328	1 887	6 178	0	0	0	0	42 487	1.97	2.09
Temporal lobe left (mm^3^)	328	4 418	12 311	0	0	0	0	74 049	4.23	2.09
Frontal lobe right (mm^3^)	328	8 565	17 036	0	0	127	8 893	99 580	6.32	2.09
Occipital lobe right (mm^3^)	328	1 003	4 688	0	0	0	0	43 885	1.43	2.09
Parietal lobe right (mm^3^)	328	5 110	13 459	0	0	0	1 041	77 898	3.84	2.09
Temporal lobe right (mm^3^)	328	7 616	16 823	0	0	0	2 755	93 323	6.43	2.09
Tumor size (mm^3^)	328	50 968	40 914	305	22 024	41673	70 917	264 510	28.80	2.09
ASA (II + III)	333	44.74%							2.45	0.60
Comorbidity	335	47.76%							2.32	0.00
Corticosteroid use	335	58.81%							4.02	0.00
HADS combined	317	0.00	1.83	−2.67	−1.32	−0.29	1.33	5.47	1.10	5.37
Presents with loss of consciousness/AED	335	51.64%							3.49	0.00
Presents with attention, executive, memory, and/or behavioral problems	335	22.39%							1.59	0.00
Presents with language problems	335	15.52%							1.68	0.00
Presents with motor deficits	335	23.28%							2.01	0.00
Presents with headache	335	23.28%							1.54	0.00

*Note*: AED = antiepileptic drugs; ASA = American Society of Anesthesiology; HADS = anxiety and depression scale.

### Model Performance

**Figure 2.**
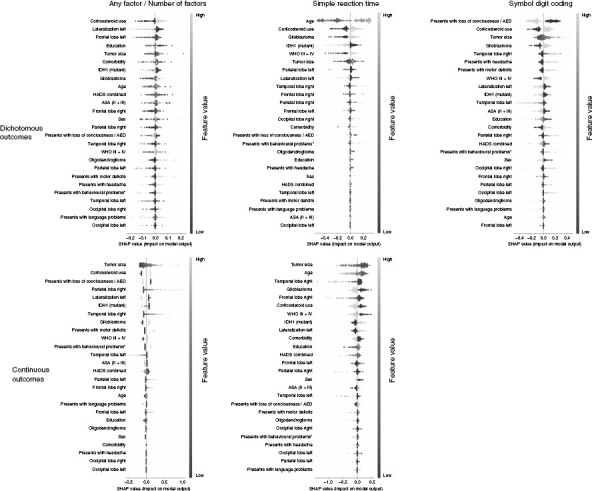
SHapley Additive exPlanations (SHAP) values for the best-performing models that provide reliable predictions. SHAP values represent the contribution of each variable given its value to the model prediction. Variables are shown in order of importance from most influential (top) to least influential (bottom). Datapoints per variable represent the values on this variable for different patients, ranging from low (dark) to high (light). Negative SHAP values (left) indicate that a variable given its value is associated with a lower test score (for continuous outcomes) or not being impaired (for dichotomous outcomes) while positive SHAP values (right) are associated with a higher test score/being impaired.

Table 3 shows the best-performing model per outcome measure as found during the double-loop cross-validation procedure. Moreover, the performance of all individual models for each of the 18 outcome measures including the optimized hyperparameters as found during the training procedure is provided as Supplementary Material to this study. Finally, SHAP values for models that provided reliable predictions are shown in Figure 2.

**Table 3.** Best-Performing Models Per Outcome Measure

			Test									Random (for reference)	
			f1/R^2^			Accuracy		Precision		Recall		f1	
	Model	Outcome measure	Mean	Std	Improvement over random	Mean	Std	Mean	Std	Mean	Std	Mean	Std
Dichotomous outcomes (f1)													
Objective 1	RandomForestClassifier	Any factor	**0.798**	**0.066**	**0.208**	0.549	0.089	0.762	0.088	0.847	0.088	0.589	0.028
Objective 3	GaussianNB	Continuous performance	0.431	0.125	0.070	0.585	0.103	0.348	0.121	0.603	0.166	0.361	0.034
	RandomForestClassifier	Finger tapping	0.427	0.123	0.059	0.584	0.087	0.391	0.134	0.494	0.149	0.368	0.034
	GaussianNB	Shifting attention	0.450	0.131	0.129	0.642	0.105	0.372	0.123	0.607	0.188	0.320	0.035
	RandomForestClassifier	Simple reaction time	**0.617**	**0.124**	**0.229**	0.730	0.084	0.569	0.149	0.723	0.178	0.388	0.027
	Elasticnet	Stroop interference	0.375	0.126	0.106	0.630	0.103	0.273	0.106	0.660	0.216	0.269	0.031
	SVC	Symbol digit coding	**0.517**	**0.115**	**0.139**	0.646	0.093	0.461	0.134	0.629	0.166	0.379	0.035
	XGB Linear	Verbal memory recognition	0.356	0.135	0.076	0.596	0.107	0.265	0.124	0.606	0.203	0.280	0.032
	KNeighborsClassifier	Visual memory recognition	0.427	0.114	0.088	0.615	0.076	0.353	0.119	0.589	0.147	0.339	0.028
Continuous outcomes (*R*^2^)													
Objective 2	ElasticNet	Number of factors	0.139	0.150									
Objective 4	ElasticNet	Continuous performance	0.005	0.085									
	KNeighborsRegressor	Finger tapping	−0.009	0.089									
	XGB Linear	Shifting attention	0.051	0.094									
	RandomForestRegressor	Simple reaction time	**0.163**	**0.138**									
	PLS Regression	Stroop interference	−0.026	0.081									
	XGB Linear	Symbol digit coding	0.078	0.126									
	ARDRegression	Verbal memory recognition	0.019	0.114									
	XGB Linear	Visual memory recognition	0.037	0.091									

*Note*: Scores indicating a performance that a model provided reliable predictions as per definition are displayed in bold. For dichotomous outcomes, the performance of the baseline model is provided for reference. For continuous outcomes, the performance of the baseline model is always 0 (*R*^2^).

#### Objective 1: Predicting impairment on at least 1 cognitive test.—

Several models obtained reliable performance as per definition for this objective. The best performance was obtained by the Random Forest model with an f1 score of 0.80 ± 0.07 (vs 0.58 resulting from the baseline model) and accuracy, precision, and recall of respectively 0.55, 0.76, and 0.85. Other good-performing models were the Gaussian process, XGB Tree, and K-nearest neighbor classifier.

The random forest is a nonlinear model that can capture interaction effects. Hyperparameter as optimized by the training procedure, however, restricted the model to using only 1 variable per tree. The model, therefore, was unable to capture interaction effects. Hyperparameters further configured the model to average over 300 trees, allowing it to consider many variables for prediction. This is confirmed by the SHAP values which show that the model relied on all variables without relying strongly on any specific variables.

SHAP values showed that the use of corticosteroids was the most important variable where using corticosteroids was associated with predicting impairment on at least 1 of the tests. This was followed by the associations of a right-lateralized tumor and the tumor not being located in the left frontal lobe with impairment on at least 1 test.

#### Objective 2: Predicting the number of impaired cognitive test scores.—

The best average performance was obtained by Elasticnet (*R*^2^ of 0.14 ± 0.15). This model, however, did not obtain reliable performance. The XGB Linear model did obtain reliable performance while having almost equivalent performance with an *R*^2^ of 0.14 ± 0.13. Therefore, we interpret the results of the XGB Linear model. Furthermore, almost equivalent performances were obtained by the Bayesian ridge and Gaussian process models.

The XGB Linear model is a linear model and is therefore unable to capture nonlinearities and interaction effects. Hyperparameter optimization selected an alpha (L1/lasso regularization) of 0.1 and a lambda (L2/ridge regularization) of 0.5. This causes the model to penalize the magnitude of coefficients while regularizing the number of non-zero variables only to a lesser extent. SHAP values confirm this and show that the model relied on 12 out of the 26 variables without relying strongly on any specific variable.

SHAP values for the XGB linear model showed that having a larger tumor was the most important variable that was associated with being impaired on more tests. This is followed by using corticosteroids and not presenting with loss of consciousness/ using an AED.

#### Objective 3: Predicting impairment separately for each cognitive test.—

For all 8 cognitive tests, best-performing models performed better than the baseline model on average with improvements in f1 scores ranging between 0.06 and 0.23. Reliable performance was found when predicting impairment on Simple reaction time and the Symbol digit coding test. For the other 6 cognitive test scores the standard deviation between train-test splits was higher than the improvement over the baseline model for all models.

**Figure 3.**
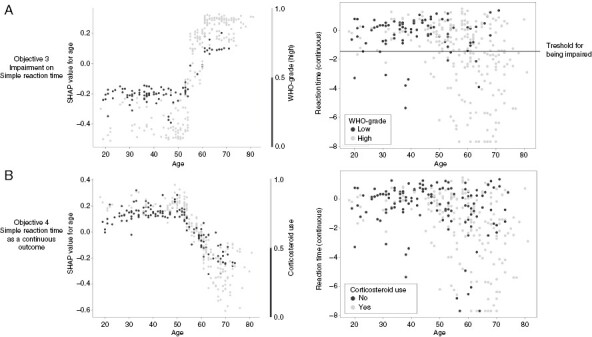
Visualization of the interaction effects as found by the models predicting impairment on the measure of Simple reaction time (**A**) and when predicting Simple reaction time as a continuous outcome (**B**). The left image shows the effect of age on the model output (y) versus age itself (x), colored by WHO grade or Corticosteroid use for figures **A** and **B**, respectively. The right image shows the measure of reaction time (y) versus age (x) colored by WHO grade or corticosteroid use for figures **A** and **B**, respectively.

When predicting impairment on the measure of Simple reaction time, the RandomForestclassifier performed best with an f1 score of 0.62 ± 0.12 (vs 0.39 resulting from the baseline model) and an accuracy, precision, and recall of 0.37, 0.57, and 0.73, respectively. Hyperparameter optimization configured the model to use a maximum tree depth of 2 while averaging over 20 models. This allows the model to find interaction effects between at most 2 variables at a time while potentially using many variables. SHAP values show that the model relied on almost all variables when making predictions without relying strongly on any specific variable. SHAP values further show that the model captured only 1 notable interaction effect where having a high WHO grade magnified the effect of increasing age. A visualization of this effect is shown in Figure 3A.

SHAP values showed that age was the most important predictor which was associated with impairment on the Simple reaction time test. The second and third most important predictors associated with impairment were using a corticosteroid and having a glioblastoma.

For predicting impairment on the Symbol digit coding test, the Support vector classifier performed best with an f1 score of 0.52 ± 0.12 (vs 0.38 resulting from the baseline model) and an accuracy, precision, and recall of 0.65, 0.46, and 0.63, respectively. Hyperparameter optimization configured the model to use a sigmoid kernel, allowing the model to capture nonlinearities and interaction effects. Hyperparameter optimization further set an L2 (ridge) penalty of 20, causing the model to rely weakly on many features. SHAP values confirm this and show that the model uses all variables for prediction without relying strongly on any specific variable. The SHAP values, however, did not show any notable interaction effects.

SHAP values for the Symbol digit coding test showed that presenting with loss of consciousness/using an AED was the most important variable which was associated with not being impaired on this test. This was followed by using a corticosteroid and having a larger tumor which were associated with being impaired on this test.

*Objective 4: Predicting cognitive function as a continuous outcome separately for each of the 8 cognitive tests.—*The explained variance for the different cognitive tests ranged between −0.03 and 0.16. Reliable performance, however, was only found when predicting Simple reaction time. For the other 7 cognitive test scores models consistently performed worse than random.

When predicting Simple reaction time, the best-performing model was the Random Forest model with an *R*^2^ of 0.16 ± 0.14. Hyperparameter optimization configured the model to use a maximum tree depth of 5 while using at most 4 variables and averaging over 20 models. This allows the model to capture interactions between 4 variables per tree. SHAP values show that the model relied on all variables for prediction without relying strongly on any specific variable. SHAP values further show 1 notable interaction effect where the use of corticosteroids in combination with older age was associated with a worse score which is shown in Figure 3B.

SHAP values for the random forest model predicting simple reaction time showed having a smaller tumor was the most important variable which was associated with better performance on this objective. This was followed by being younger and not having a tumor in the right temporal lobe.

## Discussion

Our extensive empirical prediction study found that predictions were reliable for only 4 out of the 18 cognitive outcome measures. For the other 14 outcome measures, predictions were unreliable. Therefore, we conclude that preoperative cognitive functioning cannot be reliably predicted across cognitive tests using the comprehensive set of clinical variables included in the current study. Furthermore, our results showed that best-performing models tend to be relatively simple with few interaction effects and that they used most of the variables for prediction while not relying strongly on any specific variable.

Reliable performance (as defined in the section “Interpretation”) was only obtained when predicting if the patient is impaired on at least 1 cognitive test (Objective 1), when predicting impairment on the measure of simple reaction time and impairment on the symbol digit coding test (Objective 3), and when predicting simple reaction time as a continuous outcome (Objective 4). For all other 14 outcome measures, the best-performing model did not obtain reliable performance. Therefore, we conclude that the variables used in this study do not allow for reliable predictions of cognitive functioning across cognitive tests, despite many of these variables being significantly related to cognitive function in previous studies. It is important to restate the models in this study including histological diagnosis, IDH mutation status, and tumor grade, which can merely be *estimated* preoperatively,^44^ potentially further limiting the accuracy of predictions when applied in clinical practice.

Of the 3 models that could model interaction effects, only 2 models captured an interaction effect that we deemed notable. Best-performing models being relatively simple with few to no notable interaction effects indicates that more complex relationships either do not exist or are too weak to find given the current sample size. Likely, such relationships could not be found due to the limited sample size and the complexity of the prediction tasks. The observation that all trained models rely on most variables while not relying strongly on any specific variable, strongly suggests that a multi-parametric (ie holistic) view of individual patients is necessary to explain variation in cognitive functioning.

Although most models rely on many predictors, we identified 5 variables that repeatedly were among the top 3 most influential predictors according to the SHAP values. These 5 variables are oligodendroglioma, glioblastoma, age, corticosteroid use, and presenting with loss of consciousness/AED use. All these 5 variables had a correlation of at least 0.32 with both WHO grade and IDH1 mutation status and had a correlation of at least 0.17 with one another. Their predictive value may thus, in part, be due to their associations with WHO grade and/or IDH1 mutation status.

The SHAP values found in this study generally showed that a lower age was associated with better performance. This effect was found while the cognitive test scores were corrected for effects of age as found in a healthy population. This may indicate that the performance of older patients was more affected by brain injury than in younger patients. Younger patients may have more cognitive reserve and/or neuroplasticity, allowing them to better compensate for the damage inflicted by the tumor.^54^ The model predicting impairment on the measure of Simple reaction time additionally captured an interaction effect between age and tumor grade as shown in Figure 3A. For patients with a higher-grade tumor and a higher age, the chance of being impaired on this task increased more strongly when compared to patients with a lower-grade tumor. This stronger effect of age for higher-grade tumors is likely the result of the higher lesion momentum of faster-growing tumors, that is, inflicting more damage in a shorter amount of time,^55^ leaving less room for neuroplastic processes. A similar, yet weaker and more difficult to explain, interaction effect was found for age and corticosteroid use when predicting Simple reaction time as a continuous outcome. It is important to restate that relationships as found by machine learning models are not per definition significant when tested using statistical models.

We identified 2 reasons that may explain the limited performance of prediction models of cognitive function in this study. First, only variables collected during clinical care were considered. There are, however, additional variables that are currently not routinely collected that have been related to cognitive function. These include functional and structural connectivity/network measures^56^ from either resting state fMRI,^30,57^ task-based fMRI,^58^ or diffusion-weighted MRI,^59^ and proximity of the tumor to certain white matter tracts.^60^ Furthermore, several molecular markers in addition to IDH status have been related to cognitive functioning.^8^ These, however, have not yet been collected in sufficient numbers in (our) clinical practice to include in retrospective analyses. Moreover, there likely are other underlying predictive factors or representations thereof that may influence cognitive functioning which have not yet been identified in previous research.

Second, predictors in this work also affect treatment decisions which may in turn affect cognitive function in the opposite direction. For example, high intracranial pressure due to mass effects negatively affects cognitive function and is often treated with corticosteroids. The use of corticosteroids thus may indicate problems with cognitive function, while also alleviating these same problems. In this study, preoperative corticosteroid use was associated with worse cognitive performance as indicated by the SHAP values.

The current sample was collected as part of clinical care and does not include patients who were unable to undergo neuropsychological testing, for instance, due to severe motor problems or cognitive deficits, or because of urgently scheduled surgery. This may have caused a slight overestimation of cognitive performance in our current sample. Moreover, cognitive functioning in this study was measured using a brief computerized test battery which does not measure free memory recall, language function, or visuoconstructive abilities, and performances may in part be dependent on processing speed.^61^ More detailed and lengthy cognitive investigations, however, are in general not part of routine preoperative clinical care for patients with brain tumors. Consequently, the current study does not exclude the possibility that there *exist* domains for which cognitive functioning is easier to predict.

We believe our results to be relevant for both clinicians and researchers as they show that the value of clinical variables may be limited when predicting cognitive functioning, despite many of these variables themselves being significantly related to cognitive function in previous studies. Therefore, clinicians should be mindful to not infer the cognitive functioning of patients from any of the variables included in this study or combinations thereof. Moreover, our results illustrate the importance of the distinction between explanatory modeling, in which one aims to find evidence for a hypothesis regarding a theoretical construct, and predictive modeling, where the goal is to predict an output value at the measurable level for a new observation. Last, our results show that researchers performing predictive modeling should at least consider using regularized linear regression models (Ridge/Lasso/ElasticNet) as well as the Random forest model.

Our results exemplify the need to collect large cross-center multimodal data sets including similar variables, imaging sequences, and measures of cognitive functioning. Large multimodal and cross-center data sets may allow for machine learning models to find (potentially more complex) relationships among the large number of variables resulting from different modalities. This may, in turn, allow for better predictions of cognitive performance. Larger data sets also allow statistical models to detect weaker and more complex relationships. Last, using cross-center data sets may allow statistical and machine learning models may generalize beyond one center.

This need for standardization and data sharing across centers has also been emphasized in a recent meta-analysis of studies assessing longitudinal cognitive outcomes of patients with a glioma^62^ and in reviews describing noninvasive methods for survival prediction^63^ and radiomics for precision medicine in patients with glioma.^64^ We do acknowledge the many challenges that are likely associated with such an endeavor. Furthermore, it is important to note that single-center research remains relevant, as the convergence of independent findings resulting from a heterogeneity of data sets and approaches is essential to ensure that we draw correct conclusions.

Future research could predict cognitive functioning *after surgery* using variables that are available before surgery. Additionally, researchers could predict functional outcomes that are more personally relevant for patients’ daily functioning, such as the ability to return to work or resume childcare activities. Furthermore, future research should continue hypothesis-driven explanatory research to improve the understanding of causal mechanisms behind cognitive impairments, which in turn helps to develop better prediction models.

## Conclusion

Preoperative cognitive functioning could not be reliably predicted across cognitive tests using the comprehensive set of clinical variables included in the current study. Therefore, clinicians should not infer the cognitive functioning of individual patients from any of these variables or combinations thereof. Our results indicate that a multi-parametric (ie holistic) view of individual patients is necessary to explain differences in cognitive functioning and stress the need to collect larger cross-center and multimodal data sets to explain and predict cognitive functioning across domains. Moreover, we hope the current study helps to solve the conflation between explanatory and predictive modeling, stimulates cross-center collaboration and standardization, and serves as a stepping stone toward predicting cognitive functioning after surgery.

## Supplementary Material

noad221_suppl_Supplementary_Appendix

noad221_suppl_Supplementary_Data_S1

noad221_suppl_Supplementary_Data_S2

## Data Availability

Data described in this work is not publicly available to protect the privacy of patients. All code used in this study is available as Supplementary Material.
