# Supplementary material for: Cognitive functioning in untreated glioma patients: The limited predictive value of clinical variables
Source: Neuro Oncol. 2023 Dec 1;26(4):670–83. doi: 10.1093/neuonc/noad221 (PMC10995520; doi:10.1093/neuonc/noad221)
Supplement: noad221_suppl_Supplementary_Appendix [file noad221_suppl_supplementary_appendix.docx]

# Appendix 1: Formulas used to obtain the cognitive domains

| Name | Description | Formula |
| --- | --- | --- |
| Verbal memory recognition | Memory recognition for words. Fifteen words are presented one at a time. The subject identified the presented words amongst new words. The immediate condition is at the beginning of the test battery and the delayed condition is at the end. | Number of items correct (immediate and delayed recall) |
| Visual memory recognition | Memory recognition for abstract images. Fifteen images are presented one at a time. Subjects identified the presented images amongst new images. The immediate condition is at the beginning of the test battery and the delayed condition is at the end. | Number of items correct (immediate and delayed recall) |
| Symbol digit coding | Eight symbols are presented on the screen with a corresponding number. Given a row of eight randomly ordered symbols, the participant is asked to provide the matching number for two minutes straight. | Correct responses - incorrect responses |
| Simple reaction time | The subject presses the space bar when a word is presented. | Average reaction time |
| Stroop test interference | The subject presses the space bar if a word is presented and the color of the word does/or does not match its semantic meaning for the congruent and incongruent trials respectively | (Average reaction time on correct responses for the incongruent trials - Average reaction time on correct responses for the congruent trials) / Average reaction time on correct responses for the congruent trials |
| Shifting attention task | A red circle and a blue square are presented on the screen. Given a third shape, the participant needs to match the shape either by color or shape. | Correct responses - incorrect responses |
| Continuous performance test | The subject responds to a target letter amongst distractors for 5 minutes straight. | Average reaction time to the target letter |
| Finger tapping test | The participant presses the spacebar as often as possible within ten seconds. This task is performed three times for each hand. | The average number of presses across left and right trails |

Caption: Formulas used to obtain the cognitive domains

# Appendix 2: Imputing IDH status

The majority of the machine-learning models evaluated in this study are unable to handle missing values. For models that are unable to handle missing values, missing values need to be imputed before training the model. In the current study, imputation is performed using multiple imputation, where each missing variable is estimated other variables.

For patients aged 55 or higher with a grade IV glioblastoma, missing values on IDH mutation status were imputed based on the literature instead of using multiple imputation. This was done as for this subset of patients, the IDH mutation status is known to be wildtype in 96% of cases^1^. Not imputing these values based on prior knowledge implies that the iterative imputer must learn how to predict IDH status for this subset of patients. The imputer would have to learn how to impute these values from the relatively small number of patients age 55 or higher with a grade 4 glioblastoma patients for whom IDH status is available. Imputing these values using the iterative imputer, therefore, likely would have resulted in less accurate estimations of IDH status when compared to imputing these values based on the literature. The more accurate the predictions of IDH status are, the more likely it is that we can accurately predict cognitive functioning. Therefore, we opted to impute these values based on clinical knowledge.

Note that the goal of imputation differs between predictive modeling and explanatory modeling. In predictive modeling, imputation is done with the goal of obtaining the highest model performance possible. For explanatory modelling, imputation is done with to goal being able to use participants with missing values without biassing the results of statistical tests.

# Appendix 3: Segmentation models

All anatomical MRI (T1, T1 contrast, T2, Flair) scans were registered to MNI space using affine transformation. Registration was performed using Regaladin^2^ from the NiftyReg package which has been shown to perform well for patients with a primary brain tumor^3^. Skull stripping was performed using HD-BET which is designed to be robust to a variety of different lesions^4^. Tumor volume was defined as the FLAIR enchancing part for low-grade gliomas and the T1 contrast enhancing part for high-grade gliomas.

Two different models were used for segmentation and the best segmentation was selected manually for each patient. Models used were nnU-Net^5^ as trained on T1, T1c, T2, and Flair scans from the BraTS dataset or subsets thereof^6,7^ and AGU-Net as available in the Radionics tool using T1c images for high-grade gliomas and FLAIR images for low-grade gliomas^8^. All automatic segmentations were manually validated and incorrect segmentations were redone semi-automatically using the snake tool in ITK-Snap^9^.

# Appendix 4: Training procedure

The models predicting impairment were optimized for and evaluated according to their f1 score (harmonic mean between precision and recall) which is insensitive to class imbalance as it does not rely on the number of true negatives. This is necessary as the majority of the patients were not impaired on individual tests (objective 3) while the majority were impaired on at least one of the tests (objective 1). Additionally, accuracy, precision, and recall were reported. Models predicting cognitive function as a continuous outcome or the number of tests on which a patient is impaired were optimized for and evaluated according to the amount of variance explained (R2).

Repeated nested cross-validation was used to obtain robust and unbiased performance estimates while trying different hyperparameters ^10^. The repeated nested cross-validation used 10 repeats where each repeat used a 10-fold train-test split. For each model, we report the mean and standard deviation of the performance based on the individual runs within the nested cross-validation. Finally, the best-performing model for each cognitive domain is trained on the complete sample to obtain a final model including the optimized hyperparameters that can be used for model interpretation.

The following steps were performed within each iteration of the cross-validation loop. First, predictors and continuous outcome variables were normalized with zero mean and unit standard deviation based on the statistics of the training data. Second multiple imputation was performed on the predictors if the model was not able to handle missing data. Multiple imputation predicts missing values in the set of predictors using all other predictors iteratively until convergence. Multiple imputation was fitted only on the training data and applied to both the train and test set. Third, minority oversampling was performed for objectives 1 and 3 during training as only a minority of patients were impaired on individual domains while being impaired on at least one of the domains, leading to a class imbalance in these objectives. Fourth, a 6-fold hyperparameter search is performed on the training set to find the best model parameters. Last, the best-performing model resulting from the hyperparameter search is trained on the complete training set. After model training, the train and test accuracy were calculated on the imbalanced train and test set using the model trained on the complete training set. This process is repeated 10 times for 10 train-test splits, resulting in 100 train and test scores for each model. Preprocessing and hyperparameter selection was performed within the cross-validation loop to prevent leakage of information from the test set to the training set.


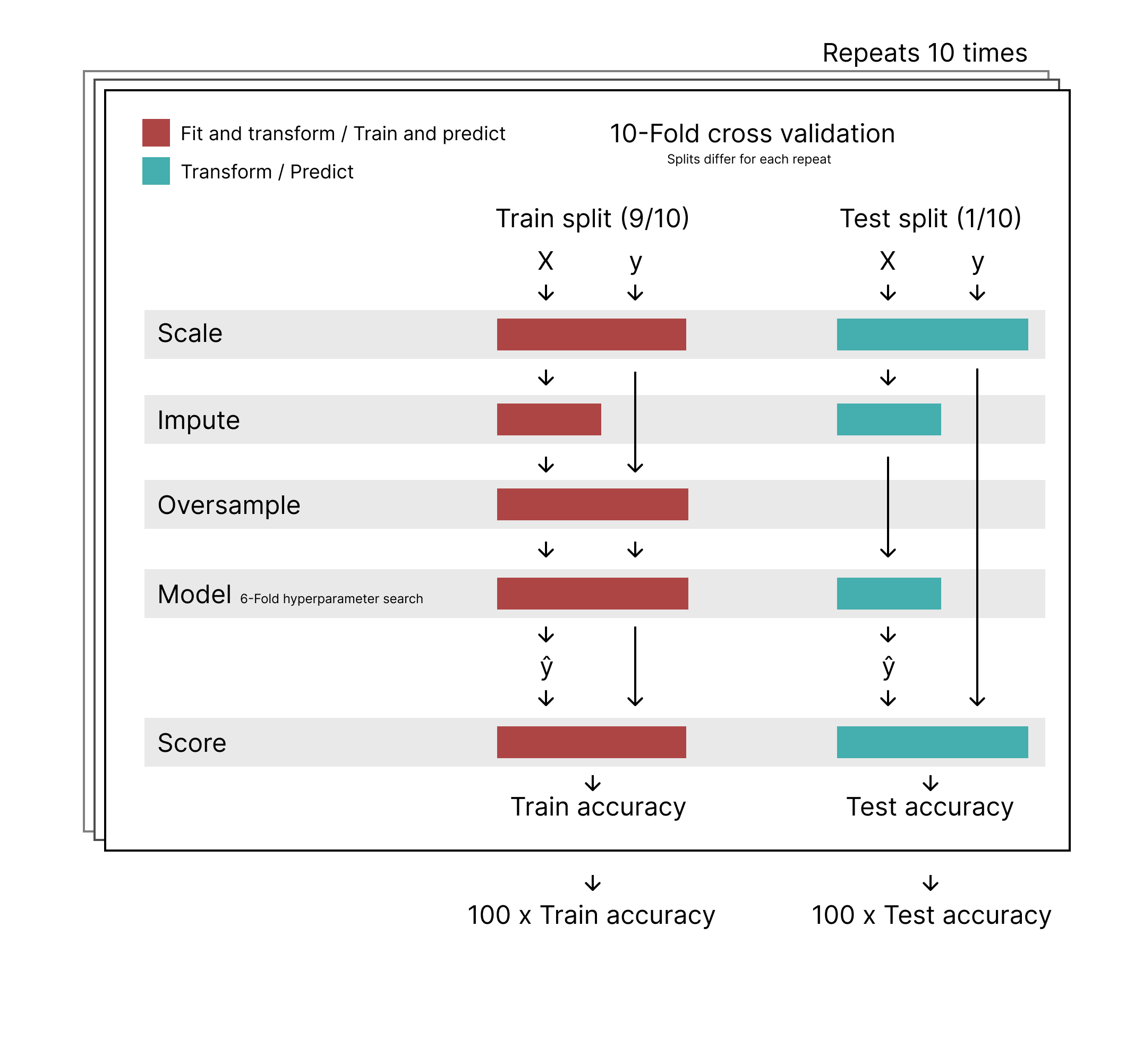

Caption: Visualization of the nested cross-validation loop

# Appendix 5: Machine learning models

Models evaluated were (logistic)regression, regularized (logistic)regression (ElasticNet), Gaussian processes regression/classification, Bayesian ridge regression, Bayesian ARD regression, K-nearest neighbor regression/classification, Decision tree regression/classification, Random forest regression/classification, Support vector regression/classification, XGB tree regression/classification, XGB linear regression/classification, Partial least squares regression, and Gaussian mixture model classification.

| Type | Model | Non-linear | Interactions between predictors | Requires imputation | Hyperparameter search |
| --- | --- | --- | --- | --- | --- |
| Regression | XGB Tree | Yes | Yes | No | "eta": [0.2, 0.4], "learning_rate": [0.01, 0.3], "max_depth": [2, 5], ‘alpha': [0.2, 0.8], ‘lambda': [0.2, 0.8] |
|  | XGB Linear | No | No | No | ‘alpha': [0.01, 0.1, 0.5, 1], ‘lambda': [0.01, 0.1, 0.5, 1] |
|  | Linear regression | No | No | Yes | None |
|  | Elasticnet | No | No | Yes | ‘alpha': [0.01, 0.1, 1, 10, 100], ‘l1_ratio': [0, 0.01, 0.1, 0.5, 0.9, 1] |
|  | Gaussian process | Yes | Yes | Yes | "kernel": [1 * RBF(0.01, length_scale_bounds=(1, 1e10)) + WhiteKernel(noise_level_bounds=(1e-10, 1e10)), 1 * RBF(0.1, length_scale_bounds=(1, 1e10)) + WhiteKernel(noise_level_bounds=(1e-10, 1e10)), 1 * RBF(0.001, length_scale_bounds=(1, 1e10)) + WhiteKernel( noise_level_bounds=(1e-10, 1e10))], "alpha": [0.1, 0.7, 1, 3, 10] |
|  | Bayesian Ridge | No | No | Yes | "alpha_1": [1e-1, 1e-3, 1e-6, 1e-18], "alpha_2": [1e-1, 1e-3, 1e-6, 1e-18], "lambda_1": [1e-1, 1e-3, 1e-6, 1e-18], "lambda_2": [1e-1, 1e-3, 1e-6, 1e-18], |
|  | ARD | No | No | Yes | "alpha_1": [1e-1, 1e-3, 1e-6, 1e-18], |
|  | K-nearest neighbor | No | No | Yes | ‘n_neighbors': [1, 2, 5, 10, 30, 60, 150] |
|  | Decision tree | Yes | Yes | Yes | ‘max_depth': [2, 5, 8, 10, 15], ‘min_samples_leaf': [1, 15, 40], ‘max_features': [1, 4, 8, None] |
|  | Random forrest | Yes | Yes | Yes | ‘max_depth': [2, 5, 8, 10, 15], ‘n_estimators': [5, 20, 100, 300], ‘min_samples_leaf': [1, 15, 40], ‘max_features': [1, 4, 8, None] |
|  | Support Vector | Yes | Yes | Yes | ‘kernel': ['linear', 'poly', 'rbf', 'sigmoid'], ‘C': [0.0001, 0.01, 0.5, 0.9, 10, 30], ‘epsilon': [0, 0.1, 1, 10] |
|  | Partial Least Squares | No | No | Yes | ‘n_components': [1, 2, 3, 4, 8, 15] |
| Classification | XGB Tree | Yes | Yes | No | "booster": ['gbtree'], "tree_method": ['gpu_hist'], "gpu_id": [1], "eta": [0.2, 0.4], "learning_rate": [0.01, 0.3], "max_depth": [2, 5], ‘alpha': [0.2, 0.8], ‘lambda': [0.2, 0.8] |
|  | XGB Linear | No | No | No | ‘alpha': [0.01, 0.1, 0.5, 1], ‘lambda': [0.01, 0.1, 0.5, 1] |
|  | Gaussian process | Yes | Yes | Yes | "kernel": [1 * RBF(0.01, length_scale_bounds=(1, 1e10)) + WhiteKernel(noise_level_bounds=(1e-10, 1e10)), 1 * RBF(0.1, length_scale_bounds=(1, 1e10)) + WhiteKernel(noise_level_bounds=(1e-10, 1e10)), 1 * RBF(0.001, length_scale_bounds=(1, 1e10)) + WhiteKernel(noise_level_bounds=(1e-10, 1e10))] |
|  | Gaussian naïve bayes | No | No | Yes | None |
|  | Logistic regression | No | No | Yes | None |
|  | K-nearest neighbor | Yes | Yes | Yes | ‘n_neighbors': [1, 2, 5, 10, 30, 60, 150], ‘weights': ['uniform', 'distance'] |
|  | Decision tree | Yes | Yes | Yes | ‘max_depth': [2, 5, 8, 10, 15], ‘min_samples_leaf': [1, 15, 40], ‘max_features': [1, 4, 8, None] |
|  | Random forrest | Yes | Yes | Yes | ‘max_depth': [2, 5, 8, 10, 15], ‘n_estimators': [5, 20, 100, 300], ‘min_samples_leaf': [1, 15, 40], ‘max_features': [1, 4, 8, None] |
|  | Support Vector | Yes | Yes | Yes | ‘kernel': ['linear', 'poly', 'rbf', 'sigmoid'], ‘C': [0.01, 0.05, 0.1, 0.5, 0.9] |

Caption: Models including hyperparameters used during the hyperparameter search, their ability to capture non-linearities and interaction effects, and their ability to handle missing data.

# Appendix 6: Description of the models used including pros and cons of each model.

In the following table, a description is provided for each model used in the current study. Moreover, pros and cons of each model are discussed. It is important to note that these pros and cons can only be interpreted in the context of the current dataset and our prior knowledge about the problem. This follows from the ‘no free lunch’ theorem which sates that all optimization problem strategies perform equally well when averaged over all possible problems^11^.

| Model | Model type | Description |  |
| --- | --- | --- | --- |
| XGB Tree | Regression and classification | XGBoost is a gradient-boosted decision tree algorithm where trees are built sequentially. Each tree is constructed to correct the errors of the previous trees. | Pros: Can model complex interactions between multiple variables. Allows for regularization. Flexible.  Cons: Computationally expensive. Resulting models are difficult to interpret. Sensitive to noisy data. Prone to overfitting. |
| XGB Linear | Regression and classification | The XGB Linear model is similar to the XGB Tree model. Instead of decision trees, this model uses multiple regularized linear models. | Pros: Allows for regularization.  Cons: Computationally expensive. Resulting models are difficult to interpret. Sensitive to noisy data. |
| Linear (logistic) regression | Regression and classification | Finds a linear relationship between the predictor variables and the outcome variable | Pros: Easy to interpret. Computationally inexpensive.  Cons: No regularization. Can only fit linear relationships. Has difficulty handling multicollinearities. |
| ElasticNet | Regression and classification | A linear model with both Ridge (L2) and Lasso (L1) regularization, regularizing the magntude of coefficients and the number of non-zero coefficients respectivelly. | Pros: Easy to interpret. Computationally inexpensive. Allows for regularization. Handles multicollinearity.  Cons: Can only fit linear relationships. |
| Gausian process | Regression and classification | A bayesian model that learns a distribution of continuous functions over all possible function values. Possible function values are dependend on the choice of the kernel. | Pros: Allows for regularization. Can model non-linear relationships and interaction effect. Flexible  Cons: Complex kernel design. Computationally expensive |
| Bayesian Ridge | Regression | A linear model with Ridge (L2) regularization, regularizing the magnitude of coefficients. Implemented using Bayesian statistics | Pros: Easy to interpret. Computationally inexpensive. Allows for regularization.  Cons: Can only fit linear relationships. |
| Gaussian naïve bayes | Classification | Models each feature as following an independent Gaussian distribution within each class. It then uses Bayes' theorem to compute the posterior probabilities for each class, assigning new data points to the class with the highest posterior probability. | Pros: Very easy to interpret. Can be fit with little data. Can model non-linear relationships and interaction effects.  Cons: Assumes independence of input features. Can only capture very simple relationships. |
| Automatic Relevance Determination (ARD) | Regression | Similar to Bayesian ridge regression, but leads to sparser coefficients. | Pros: Easy to interpret. Computationally inexpensive. Allows for regularization. Results in sparse coefficients.  Cons: Can only fit linear relationships |
| K-nearest neighbor | Regression and classification | A non-parametric method that does not fit a model. For regression tasks, it assigns the average value of the *k* most similar data points to a new data point. For classification, it assigns the most frequent class among the *k* nearest neighbors. | Pros: Has no internal model which is fitted to the data (non-parametric). Works with small sample sizes. Is considerate of non-linear relationships and interaction effects.  Cons: Does not create a model, therefore there is no model which can be interpreted. Sensitive to irrelevant features. |
| Decision tree | Regression and classification | Learns a tree consisting of if-then-else decision rules based on the predictors represented as nodes in the tree. The tree makes binary decisions at each node, aiming to partition the data into subsets that are increasingly homogeneous. The leaf nodes of the tree contain the final predictions, which can be an average of the target variable for regression or the most frequent class for classification tasks. | Pros: Easy to interpret. Models non-linear relationships and interaction effect. Automatic feature selection.  Cons: Prone to overfitting |
| Random forrest | Regression and classification | An ensemble of decision trees. Multiple decision trees are fitted on different sub-samples of the data. For regression, the final prediction averages of the multiple models. For classificantion, the prediction is a majority vote between the model. | Pros: Models non-linear relationships and interaction effects. Generalizes well due to model averaging (bagging). Robust to noise. Automatic feature selection.  Cons: More difficult to interpret when compared to decision trees. |
| Support Vector Machine | Regression and classification | A model that aims to find a hyperplane that maximizes the margin between different classes in the case of classification or minimizes the error in the case of regression. Non-linear relationships can be modeled using kernel functions that map the data into a higher-dimensional space. | Pros: Can model non-linear relationships and interaction effects.  Cons: Computationally expensive. Requires kernel design. Non-linear kernels may be difficult to interpret. |
| Partial Least Squares | Regression | Partial least squares aims to find mutidimensional directions in the input variables that explain as much variance as possible in the output variable. PLS regression can be interpreted as a supervised version of performing PCA. | Pros: Works well with high dimensional data.  Cons: Does not model non-linearities and interaction effects. Resulting components may not have a straightforward interpretation. |

Caption: Description of the models used including pros and cons of each model.

# Appendix 7: VIF scores before reducing the number of variables

| Variable | VIF |
| --- | --- |
| Age | 19.77378 |
| Education | 15.83038 |
| Sex | 3.140867 |
| Glioblastoma | 12.81623 |
| Oligodendroglioma | 1.843913 |
| WHO 3 + 4 | 11.71942 |
| IDH1 (mutant) | 4.049691 |
| Lateralization left | 6.016092 |
| Frontal lobe left | 9.26952 |
| Occipital lobe left | 1.524934 |
| Parietal lobe left | 2.010252 |
| Temporal lobe left | 4.280619 |
| Frontal lobe right | 6.441537 |
| Occipital lobe right | 1.443207 |
| Parietal lobe right | 3.88374 |
| Temporal lobe right | 6.465663 |
| Tumor size | 29.13227 |
| ASA (I + II) | 2.442174 |
| Comorbidity | 2.352007 |
| Corticosteroid use | 4.151387 |
| Antiepileptic drug use | 5.565098 |
| HADS anxiety | 6.17248 |
| HADS depression | 5.429865 |
| Presents with attention, executive, memory, and/or behavioral problems | 1.63503 |
| Presents with language problems | 1.703779 |
| Presents with loss of consciousness | 5.537907 |
| Presents with motor deficits | 2.064283 |
| Presents with headache | 1.573036 |

Caption: 2:VIF scores before reducing the number of variables based on correlations and VIF scores.

# Implementation

The machine-learning pipeline used in this work was implemented using Python and used models from both Scikit-learn and XGBoost. Hierarchical clustering was implemented using the algorithm provided in Scipy. Shapley additive explanations were calculated using the SHAP library. All code is publicly available as online supplementary material to this study.

# References

1. DeWitt JC, Jordan JT, Frosch MP, et al. Cost-effectiveness of IDH testing in diffuse gliomas according to the 2016 WHO classification of tumors of the central nervous system recommendations. *Neuro-Oncology*. 2017;19(12):1640-1650. doi:10.1093/neuonc/nox120

2. Ourselin S, Roche A, Subsol G, Pennec X, Ayache N. Reconstructing a 3D structure from serial histological sections. *Image and Vision Computing*. 2001;19(1-2):25-31. doi:10.1016/S0262-8856(00)00052-4

3. Visser M, Petr J, Müller DMJ, et al. Accurate MR Image Registration to Anatomical Reference Space for Diffuse Glioma. *Front Neurosci*. 2020;14:585. doi:10.3389/fnins.2020.00585

4. Isensee F, Schell M, Pflueger I, et al. Automated brain extraction of multisequence MRI using artificial neural networks. *Hum Brain Mapp*. 2019;40(17):4952-4964. doi:10.1002/hbm.24750

5. Isensee F, Jaeger PF, Kohl SAA, Petersen J, Maier-Hein KH. nnU-Net: a self-configuring method for deep learning-based biomedical image segmentation. *Nat Methods*. 2021;18(2):203-211. doi:10.1038/s41592-020-01008-z

6. Bakas S, Reyes M, Jakab A, et al. Identifying the Best Machine Learning Algorithms for Brain Tumor Segmentation, Progression Assessment, and Overall Survival Prediction in the BRATS Challenge. *arXiv:181102629 [cs, stat]*. Published online April 23, 2019. Accessed October 7, 2020. http://arxiv.org/abs/1811.02629

7. Menze BH, Jakab A, Bauer S, et al. The Multimodal Brain Tumor Image Segmentation Benchmark (BRATS). *IEEE Trans Med Imaging*. 2015;34(10):1993-2024. doi:10.1109/TMI.2014.2377694

8. Bouget D, Pedersen A, Jakola AS, et al. Preoperative brain tumor imaging: models and software for segmentation and standardized reporting. *arXiv:220414199 [cs, eess]*. Published online April 29, 2022. Accessed May 10, 2022. http://arxiv.org/abs/2204.14199

9. Yushkevich PA, Piven J, Hazlett HC, et al. User-guided 3D active contour segmentation of anatomical structures: Significantly improved efficiency and reliability. *NeuroImage*. 2006;31(3):1116-1128. doi:10.1016/j.neuroimage.2006.01.015

10. Vabalas A, Gowen E, Poliakoff E, Casson AJ. Machine learning algorithm validation with a limited sample size. Hernandez-Lemus E, ed. *PLoS ONE*. 2019;14(11):e0224365. doi:10.1371/journal.pone.0224365

11. Gómez D, Rojas A. An Empirical Overview of the No Free Lunch Theorem and Its Effect on Real-World Machine Learning Classification. *Neural Computation*. 2016;28(1):216-228. doi:10.1162/NECO_a_00793
